# Integrating Network Pharmacology and Experimental Validation to Investigate the Effects and Mechanism of *Astragalus* Flavonoids Against Hepatic Fibrosis

**DOI:** 10.3389/fphar.2020.618262

**Published:** 2021-01-22

**Authors:** Lin An, Yuefang Lin, Leyan Li, Muyan Kong, Yanmei Lou, Jinjun Wu, Zhongqiu Liu

**Affiliations:** Joint Laboratory for Translational Cancer Research of Chinese Medicine of the Ministry of Education of the People's Republic of China, International Institute for Translational Chinese Medicine, School of Pharmaceutical Science, Guangzhou University of Chinese Medicine, Guangzhou, China

**Keywords:** flavonoids, *Astragalus membranaceus* (fisch) bunge, liver fibrosis, anti-inflammation, network pharmacology

## Abstract

Hepatic fibrosis (HF) represents the excessive wound healing where an excess amount of connective tissues is formed within the liver, finally resulting in cirrhosis or even hepatocellular carcinoma (HCC). Therefore, it is significant to discover the efficient agents and components to treat HF, thus restraining the further progression of hepatopathy. *Astragalus membranaceus* (Fisch.) Bunge [also called *Astragali* Radix (AR)] is a famous herb in traditional Chinese medicine (TCM), which possesses a variety of biological activities and exerts good therapeutic effects in the treatment of HF. Flavonoids account for the major active ingredients related to the AR pharmacological effects. Total AR flavonoids have been proved to exert inhibitory effects on hepatic fibrosis. This study aimed to further undertake network pharmacology analysis coupled with experimental validation and molecular docking to investigate the effects and mechanism of multiple flavonoid components from AR against liver fibrosis. The results of the network pharmacology analysis showed that the flavonoids from AR exerted their pharmacological effects against liver fibrosis by modulating multiple targets and pathways. The experimental validation data showed that the flavonoids from AR were able to suppress transforming growth factor beta 1 (TGF-β1)-mediated activation of hepatic stellate cells (HSCs) and reduce extracellular matrix deposition in HSC-T6 cells via regulating the nuclear factor kappa B (NF-κB) signal transduction pathway. The results of the molecular docking study further showed that the flavonoids had a strong binding affinity for IκB kinase (IKKβ) after docking into the crystal structure. The above results indicated that, flavonoids possibly exerted the anti-inflammatory effect on treating HF by mediating inflammatory signaling pathways. The potential mechanism of these flavonoids against liver fibrosis may be related to suppression of the NF-κB pathway through effective inhibition of IKKβ. This study not only provides a scientific basis for clarifying the effects and mechanism of AR flavonoids against liver fibrosis but also suggests a novel promising therapeutic strategy for the treatment of liver fibrosis.

## Introduction

Hepatic fibrosis (HF) is a pathological event where connective tissues abnormally proliferate in liver, which is associated with a variety of factors, causing excess extracellular matrix (ECM) deposition within liver (Trautwein et al., 2015). When there is acute injury, liver can recover the structural integrity of liver, in the meantime of sustaining the unfavorable chronic injury-derived stimuli, resulting in liver parenchymal distortion (Parola and Pinzani, 2019). For HF, its pathogenic mechanisms are chronic virus infection, nonalcoholic steatohepatitis (NASH), alcoholic liver disease (ALD), together with toxin-related stimuli (such as drugs and alcohol), autoimmune hepatitis, hereditary metabolic disease and cholestatic diseases (Fan et al., 2019). Such injury to liver parenchyma will activate the hepatic stellate cells (HSCs), the cells responsible for storing vitamin A and are involved in HF occurrence (Huang et al., 2017). Upon activation, HSCs gradually transform into myofibroblasts, a heterogeneous cell population with proliferating, migrating, and fibrotic characteristics. This transformation causes a continuous accumulation of alpha smooth muscle actin (α-SMA), ECM, and a large amount of type I and type III collagen, leading to scar deposition (Parola and Pinzani, 2019). In addition, HSCs can produce the transforming growth factor beta 1 (TGF-β1) to induce a fibrotic response, as well as the subsequent connective tissue proliferation (Xu et al., 2020). Cirrhosis is the end-stage disease of HF, and it stands for a major reason for the morbidity and mortality in the world (Scaglione et al., 2015). At present, liver transplantation (LT) remains the unique effective way to treat cirrhosis patients; as a result, it is significant to discover the efficient agents and components to treat HF, thus restraining the further progression of hepatopathy and preventing death. HF or even the early cirrhosis, is suggested to be reversible (Friedman et al., 2000). Certain herbs in the traditional Chinese medicine (TCM) are utilized in clinical treatments for a long history due to the efficient therapeutic effect and usability, particularly in treating hepatopathy (Xie et al., 2013). As a result, applying TCM herbs may serve as the efficient way to prevent and treat HF.

*Astragalus membranaceus* (Fisch.) Bunge is also called *Astragali* Radix (AR), and it accounts for an extensively used TCM herb in China, with a usage history of more than 2,000 years. Traditionally, AR is frequently applied to treat various diseases, including weakness, anemia, fever, chronic fatigue, wounds, loss of appetite, multiple allergies, and uterine bleeding (Guo et al., 2019). For these bioactive functions, AR has been widely used as a kind of health food additive supplemented in various drinks or in additional food forms for the effective body immunity reinforcement (Bai et al., 2018). Modern pharmacological experiments have indicated that AR has different biological activities, like antioxidation, immunomodulation, anticancer and anti-inflammation, which has been extensively utilized in treating nephritis, diabetes and cancers (Xu et al., 2006; Guo et al., 2019). AR has also been recognized to significantly protect from injuries to intestine, heart, liver, lung, kidney, and brain (Shahzad et al., 2016). Beyond that, AR is commonly used in Chinese herb prescription to treat HF in Chinese clinical practice (Ding et al., 2011; Xu et al., 2015). Modern pharmacological studies has also confirmed that AR can effectively suppress HF (Liang and Yuan, 2013; Zhou et al., 2016; Wang et al., 2019). So far, over 200 components are separated in AR. Typically, flavonoids represent the leading ingredients related to the efficacy and pharmacological effects (Guo et al., 2019). The total flavonoids from AR have been proved to exert inhibitory effects on HF (Liu et al., 2005; Li et al., 2019). However, the specific AR flavonoid components responsible for inhibition on HF, as well as the precise molecular mechanisms, are not completely understood.

TCM herbs are the effective herbal medicines adopted for treating a variety of disorders, which exert pivotal parts in healthcare in the history of China or even numerous other countries. However, it is difficult to elucidate the effects of TCMs because of their multiple targets, multiple pathways, and multiple mechanisms of action. The above features have severely restricted the internationalization and modernization of herbal medicines (Gan et al., 2019). Recently, network pharmacology has been developed to be the effective approach for observing the interactions and impacts of different drugs on disease network (Li and Zhang, 2013; Hao and Xiao, 2014). It integrates chemoinformatics, bioinformatics, network biology, network analysis, and traditional pharmacology (Berger and Iyengar, 2009). Therefore, network pharmacology provides a novel and effective tool that can combine the TCM target database in a variety of disorders and verify at molecular level, thus providing evidence regarding the molecular targets as well as possible underlying mechanisms of herbal medicines (Gan et al., 2019).

**FIGURE 1:**
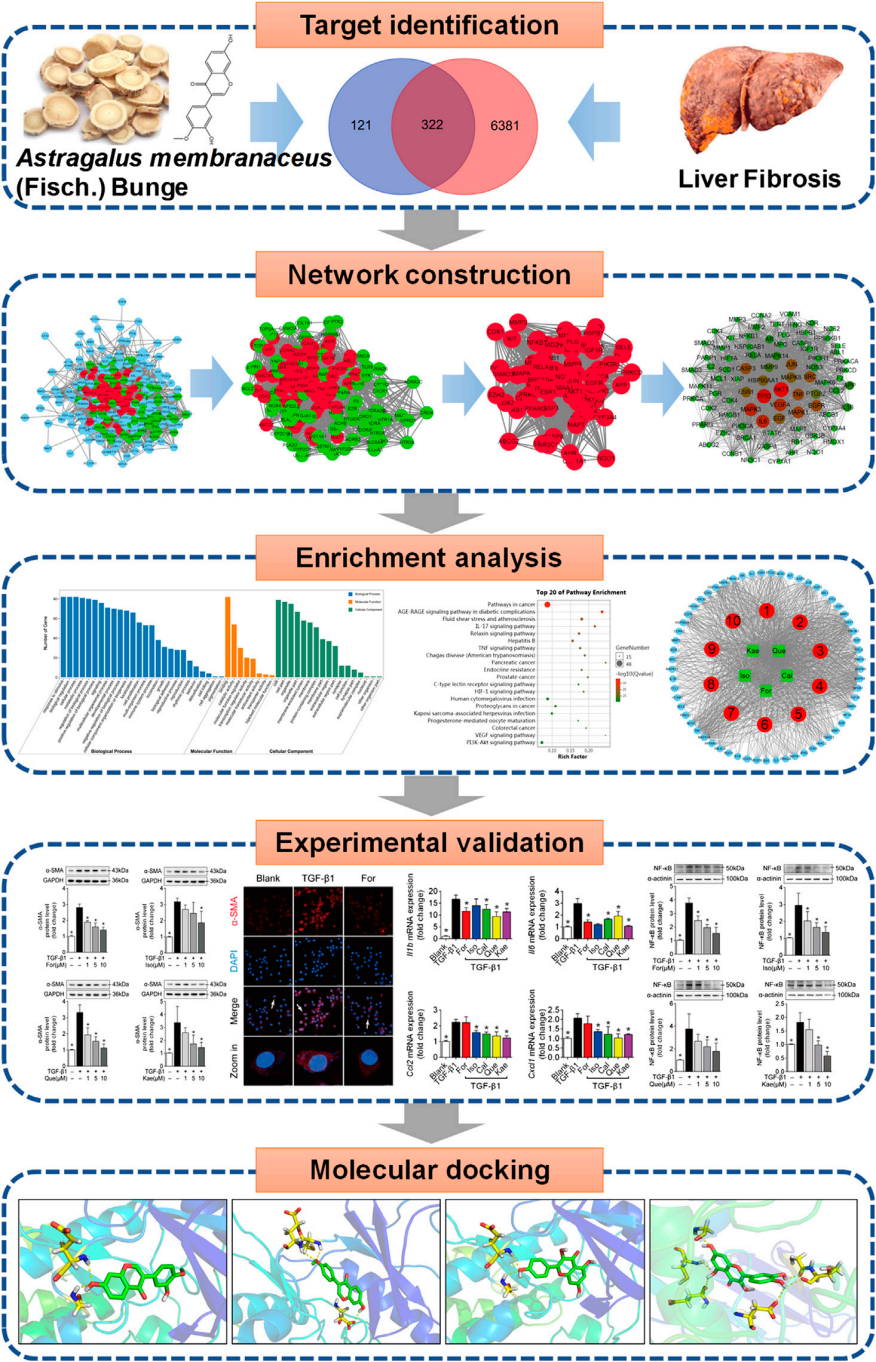
Flowchart showing the systems pharmacology approach for determining the pharmacological effects and mechanism of *Astragali* Radix (AR) in the treatment of liver fibrosis by integrating target identification, network construction, enrichment analysis, experimental validation and a molecular docking study.

Therefore, the present work applied network pharmacology in combination with experimental validation for clarifying the activities and possible mechanisms of flavonoids derived from AR to treat HF (Figure 1). The main objectives of the present study were 1) to screen the active flavonoid compounds of AR, to predict the potential targets of these flavonoids against liver fibrosis, and to analyze the potential mechanism of the flavonoids against liver fibrosis by using network pharmacology; 2) to employ TGF-β1 to stimulate HSC transdifferentiation into myofibroblasts to investigate the antifibrotic activity of the flavonoids; 3) to elucidate the potential influence of the flavonoids on HSC activation from the aspect of the inflammatory signaling pathway; and 4) to study the binding ability between flavonoids and key target genes by using molecular docking. Our results may help to understand the mechanisms of AR against liver fibrosis and suggest a novel promising therapeutic strategy for its treatment.

## Materials and Methods

### Screening for Active Flavonoid Compounds in AR

According to ADME (absorption, distribution, metabolism, excretion) parameters (Xu et al., 2012), the active flavonoid compounds of AR were screened by searching the Traditional Chinese Medicine Systems Pharmacology (TCMSP, https://tcmspw.com/tcmspsearch.php) (Ru et al., 2014), the Encyclopedia of Traditional Chinese Medicine (ETCM, http://www.ehbio.com/ETCM), and the Integrative Pharmacology-based Research Platform of Traditional Chinese Medicine (TCMIP, http://www.tcmip.cn) databases (Xu et al., 2019). Oral bioavailability (OB) > 30%, along with drug similarity (DL) > 0.18, was used for screening (Li et al., 2015).

### Prediction of the Targets of the Active Ingredients in AR

The target prediction for the main active compounds was performed by searching the TCMSP (Ru et al., 2014), PubChem (https://pubchem.ncbi.nlm.nih.gov/) (Zhu et al., 2019), STITCH (http://stitch.embl.de/) (Zhu et al., 2019), SuperPred (http://prediction.charite.de/) (Nickel et al., 2014) and Swiss Target Prediction (http://www.swisstargetprediction.ch/) (Gan et al., 2019) databases with the “*Homo sapiens*” species setting. All targets were combined and identified using the UniProt database (https://www.uniprot.org/) (Gan et al., 2019). Duplicates of the validated and predicted targets were eliminated, and the targets of the active ingredients were screened. Finally, a visual compound-target network was established with Cytoscape v3.7.2, the open-source software utilized to visualize the complicated networks and integrate all attribute data (Shannon et al., 2003).

### Screening of Potential Targets for Liver Fibrosis

The GeneCards (https://www.gene-(cards.org/) (Safran et al., 2003) and DisGeNET (http://www.disgenet.org/web/DisGeNET/menu/home) (Ren et al., 2019) databases were searched with the keywords “HF” or “liver fibrosis” to identify targets related to HF.

### Network Construction

The common targets of the flavonoid ingredients and liver fibrosis were collected. The STRING database (https://string-db.org/) was utilized to construct the measureable targets of the protein-protein interaction (PPI) network for those main components to treat HF (Szklarczyk et al., 2015). Additionally, Cytoscape was adopted for visualizing and integrating those topological parameters in PPI network regarding the main components to treat HF. The key biological targets were acquired based on the Cytoscape degree.

### Enrichment Analysis

OmicShare tools (http://www.omicshare.com/tools), the freely accessible online platform to analyze data, was utilized for Gene Ontology (GO) as well as Kyoto Encyclopedia of Genes and Genomes (KEGG) pathway enrichment analysis for the key targets. GO annotation was classified into three distinct categories, namely, biological process (BP), cell component (CC), and molecular function (MF), and such method was adopted for identifying those potential mechanisms based on the high-throughput transcriptome and genome data (Ashburner et al., 2000). In addition, the KEGG database (https://www.kegg.jp/) was used to identify the systematic functions and biological relevance of candidate targets (Chen et al., 2015). In the current study, the top 20 enriched KEGG pathways were displayed, and only terms with an FDR < 0.05 were selected for analysis. Finally, according to results of KEGG analysis, Cytoscape v3.7.2 was applied in constructing a compound-target-pathway network. The characteristics of multiple components, multiple targets and multiple pathways of the active ingredients against liver fibrosis were revealed through the construction of a network.

### Chemicals and Reagents

Formononetin (For, A0232), isorhamnetin (Iso, A0514), kaempferol (Kae, A0129), calycosin (Cal, A0190), and quercetin (Que, A0083) (purity ≥ 98%) were provided by Chengdu Must Biotechnology Co., Ltd. (Chengdu, China), which were mixed with dimethyl sulfoxide (DMSO, 0.1%, v/v; Sigma, St. Louis, MO, United States). In the meantime, the HEK293-derived recombinant human TGF-β1 (100-21) was purchased from PeproTech (New Jersey, United States). The anti-α-SMA (14395-1-AP) and anti-GAPDH (6004-1-lg) antibodies, together with the HRP-labeled AffiniPure goat anti-mouse IgG antibody (H + L) (SA00001-1), were provided by Proteintech (Wuhan, China). The HRP-labeled goat anti-rabbit IgG antibody (H + L) (BS13278) was provided by Bioworld Technology (St. Paul, MN, United States). The Alexa fluor 568-conjugated goat anti-rabbit IgG (H + L) antibody was purchased from Abcam (Cambridge, MA, United States). The enhanced BCA protein assay kit (P0010), RIPA lysis buffer (P0013) and DAPI staining solution (C1005) were provided by Beyotime Institute of Biotechnology (Shanghai, China).

### Cell Culture and Treatment

HSC-T6, a rat HSC line, was kindly presented by Zhejiang Cancer Hospital (Zhejiang, China) and cultured in DMEM (Thermo Fisher Scientific, MA, United States) containing 10% fetal bovine serum (FBS, Thermo Fisher Scientific, MA, United States), 100 μg/ml streptomycin and 100 U/ml penicillin. Afterward, cells were subjected to incubation under 37°C and 5% CO_2_ conditions. When cells reached confluence, PBS was used to wash cells for two times and the serum-free medium was used for further cell culture prior to subsequent experiment.

### Cell Viability Assay

Cellular viability was detected with a CCK-8 assay kit (GLPBIO, California, United States). The HSC-T6 cell line was planted into the 96-well culture plate, followed by 24 h of exposure to various doses of For, Iso, Cal, Que and Kae (0.1–200 μM). Thereafter, 10 μl CCK-8 solution was placed into all wells to incubate for 1 h, and the microplate reader (Thermo Varioskan LUX, MA, United States) was used to measure the absorbance (OD) value at 450 nm.

### Quantitative Real-Time Polymerase Chain Reaction (RT-qPCR)

The total pulmonary or cellular RNA was extracted using the TRIzol reagent (Accurate Biotechnology, Human, China) in accordance with specific instructions. Then, 1 ng RNA was used to synthesize cDNA *via* the *Evo M-MLV* RT Kit as well as the gDNA Clean to carry out RT-qPCR (Accurate Biotechnology, Human, China). We performed RT-qPCR for each cDNA sample in triplicate using the SYBR Green Premix *Pro Taq* HS qPCR kit (Accurate Biotechnology, Human, China) and gene-specific primer pairs for *Gapdh*, *Acta2*, *Fn1*, *Col1a1*, *Col3a1*, *Rela*, *Jun*, *Il1β*, *Il6*, *Tnfa*, *Ifng*, *Ccl2*, *Cxcl2*, *Cox2*, *Mmp1*, *Mmp2*, and *Mmp9* (Table 1). A QuantStudio 5 Real-Time PCR instrument (Thermo Fisher Scientific, MA, United States) was applied in PCR amplification under the conditions below: 30 s of enzyme activation under 95°C, 5 s of denaturation under 95°C, followed by 40 cycles of 30 s of annealing and extending under 60°C. In this study, those semi-quantitative RT-qPCR data of every target gene were expressed as 2^−*ΔΔ*Ct^ relative expression compared with endogenous GAPDH, and the error bar represented standard error of the mean (SEM) from three independent experiments.

**TABLE 1:** Nucleotide sequences of the gene-specific primers used for RT-qPCR.

Species	Primers	Sequences
*Rattus norvegicus*	Gapdh-forward	AGT​GCC​AGC​CTC​GTC​TCA​TA
	Gapdh-reverse	GAT​GGT​GAT​GGG​TTT​CCC​GT
	Acta2-forward	CAT​CCG​ACC​TTG​CTA​ACG​GA
	Acta2-reverse	GTC​CAG​AGC​GAC​ATA​GCA​CA
	Fn1-forward	CACCGAAACCGGGAAGAG
	Fn1-reverse	TTG​CCT​AGG​TAG​GTC​CGT​TC
	Col1a1-forward	GTGCGATGGCGTGCTATG
	Col1a1-reverse	ACT​TCT​GCG​TCT​GGT​GAT​ACA
	Col3a1-forward	AGA​TGC​TGG​TGC​TGA​GAA​GAA​AC
	Col3a1-reverse	GCT​GGA​AAG​AAG​TCT​GAG​GAA​GG
	Rela-forward	CTG​GCC​ATG​GAC​GAT​CTG​TT
	Rela-reverse	TCC​ACA​TAT​GGC​CCA​GAA​GC
	Jun-forward	TGT​CTG​TAT​GCT​GGG​GTG​A
	Jun-reverse	GGTTGCTGGGGAGAGAGA
	Il1β-forward	TGC​AGG​CTT​CGA​GAT​GAA​C
	Il1β-reverse	GGG​ATT​TTG​TCG​TTG​CTT​GTC
	Il6-forward	CAT​TCT​GTC​TCG​AGC​CCA​CC
	Il6-reverse	GCA​ACT​GGC​TGG​AAG​TCT​CT
	Tnfa-forward	CTT​CTG​TCT​ACT​GAA​CTT​CGG​G
	Tnfa-reverse	CTACGGGCTTGTCACTCG
	Ifng-forward	CATGAGCATCGCCAAGT
	Ifng-reverse	CTC​TAC​CCC​AGA​ATC​AGC​A
	Ccl2-forward	TTAAGCCCCACTCACCTG
	Ccl2-reverse	CTC​TTG​AGC​TTG​GTG​ACA​AAT​AC
	Cxcl1-forward	TCC​AGA​GTT​TGA​AGG​TGA​TGC
	Cxcl1-reverse	GGA​CAC​CCT​TTA​GCA​TCT​TTT​G
	Cox2-forward	GAT​GAC​GAG​CGA​CTG​TTC​CA
	Cox2-reverse	TGG​TAA​CCG​CTC​AGG​TGT​TG
	Mmp1-forward	AGC​TCA​TAC​AGT​TTC​CCC​GTG
	Mmp1-reverse	CCT​CCT​TGG​CAT​CCA​CGT​TT
	Mmp2-forward	TTT​GGC​CGT​CTC​TTC​CAT​CC
	Mmp2-reverse	GCA​TCG​ATC​TTC​TGG​ACG​GT
	Mmp9-forward	GAT​CCC​CAG​AGC​GTT​ACT​CG
	Mmp9-reverse	GTT​GTG​GAA​ACT​CAC​ACG​CC

### Western Blot Analysis

Cell lysates were produced within the RIPA buffer that contained the fresh phosphatase and protease inhibitors. Following 15 min of centrifugation at 13,000 rpm and 4°C, we collected supernatants to a new tube, followed by boiling within the 1% SDS loading buffer for obtaining samples. Afterward, the resultant samples were added onto the 10% SDS-PAGE, and the protein were transferred onto the NC membranes, followed by 2 h of incubation with 5% nonfat skim milk (FUJIFILM Wako Pure Chemical Corporation, Fuji, Japan) supplemented within the Tris-buffered saline that contained 0.1% Tween. Later, the membranes were incubated with anti-α-SMA and anti-p50 antibodies (1:800) overnight, washed, and further incubated by the HRP-labeled anti-rabbit or anti-mouse IgG secondary antibodies (1:10,000). ECL-enhanced chemiluminescence (Yeasen, Shanghai, China) was used to develop bands, whereas Image-ProPlus 6.0 (Rockville, MD, United States) was utilized for visualization.

### Immunofluorescence Assay

The HSC-T6 cell line was exposed to 10 ng/ml TGF-β1 for 24 h in the presence or absence of 10 μM diverse compounds. Thereafter, all cells were rinsed by the cold PBS, followed by 20 min of 4% paraformaldehyde fixation. Later, 0.2% Triton X 100 was used to permeabilize cells, followed by 5% BSA incubation for blocking the nonspecific staining as well as incubation using α-SMA antibody staining under 4°C overnight within the humid chamber. Then, cells were rinsed repeatedly before further 1 h of incubation using Alexa fluor 568-conjugated goat anti-rabbit IgG (H + L) antibody (1:200) under 37°C. Finally, PBS was used to rinse cells for two times, followed by 15 min of incubation with DAPI under 37°C. Images were viewed by confocal scanning microscopy (Leica TCS SP8, Leica, Germany) at an excitation wavelength of 568 nm.

### Molecular Docking

Nuclear factor kappa B (NF-κB) proteins are a crucial family of transcription factors in the signal transduction cascade of inflammatory signaling (Hoesel and Schmid, 2013). NF-κB activation is determined by IκB kinase (IKKβ)-mediated IκBα phosphorylation, as well as the later p50–p65 subunit nuclear translocation of NF-κB, finally inducing the expression of pro-inflammatory cytokines (Hoesel and Schmid, 2013). Therefore, the IKKβ selective inhibitor might be used as the candidate drug to resist inflammation. Accordingly, to further analyze the way in which the flavonoids of AR inhibit the inflammatory NF-κB pathway, the interactions between the flavonoids and IKKβ were further measured by AutoDock Vina software. The X-ray crystallographic structure of IKKβ was retrieved from the Protein Data Bank (PDB ID: 3RZF) (Xu et al., 2011). The 3D structures of the AR flavonoids taken as ligands were retrieved from the PubChem database. The grid box for IKKβ used centers of 92.23, −25.49, and 53.01 Å with corresponding sizes of 60, 60, and 60 (x, y, and z positions, respectively) for comprising each amino acid residue of the protein simulated. The other detailed docking procedures were performed as previously described (Ruan et al., 2020). After docking, the flavonoid ligands with the lowest affinity score for the receptor IKKβ were selected for further analysis. All the structure-based features were visualized by using PyMOL software.

### Data Analysis

Values were presented in the manner of mean ± SD from three independent replicates. GraphPad Prism 7.0 (San Diego, CA, United States) was used for all statistical analyses. Significant differences were analyzed by single-factor ANOVA and two-tailed Student’s *t*-test between the samples and their respective controls. Differences were considered statistically significant at *p*-values of <0.05.

## Results

### Active AR Flavonoid Compounds

**FIGURE 2:**
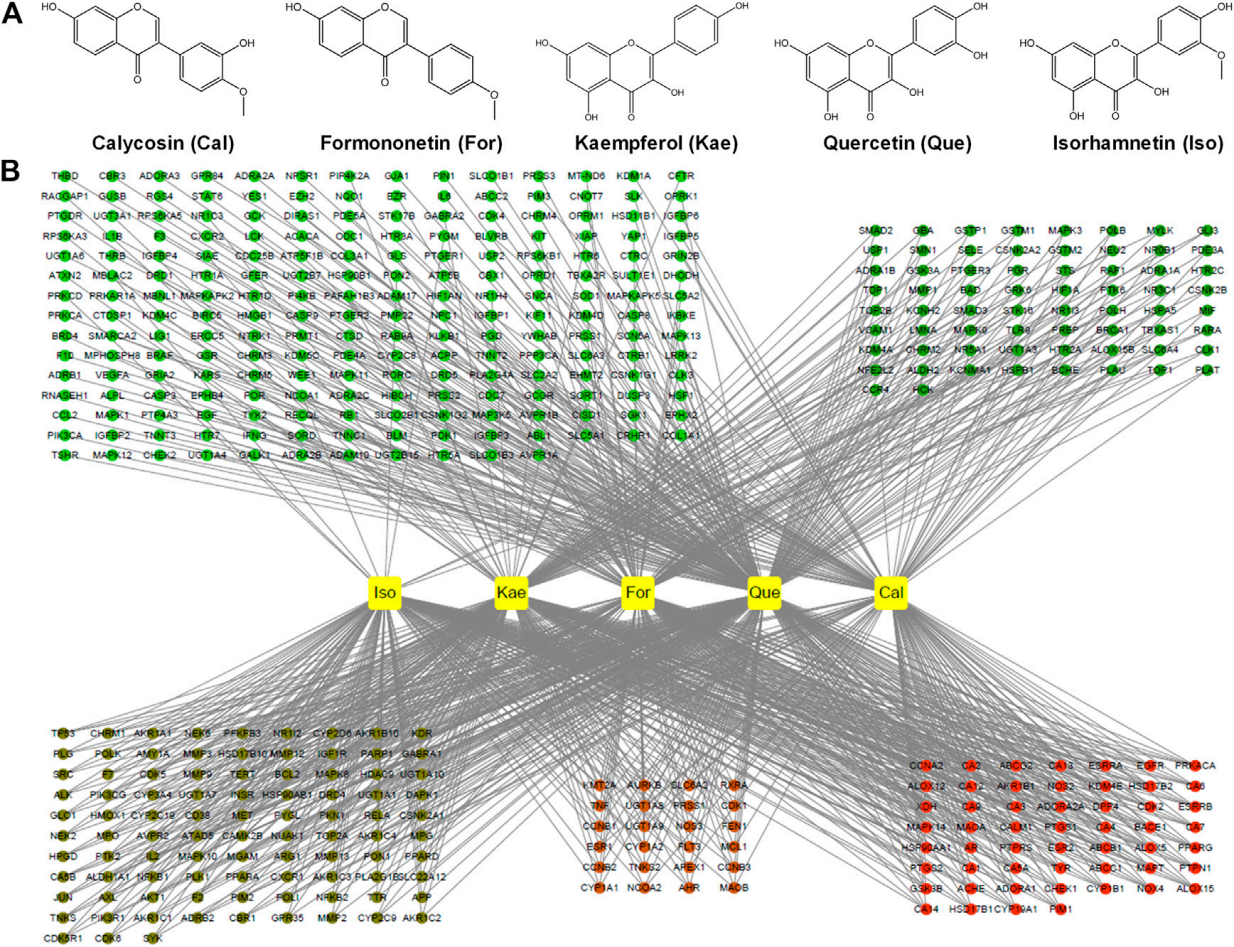
Screening for active flavonoid compounds in AR and their targets. **(A)** Molecular formulas of the five flavonoids, including For, Iso, Cal, Que, and Kae. **(B)** Compound target network (C-T network). Network of five active flavonoids of AR and 443 putative targets, among which there were 141 for Cal, 144 for Iso, 167 for, 212 for Kae and 315 for Que.

A total of 87 reported active ingredients of AR were retrieved by searching the TCMSP database. A total of 27 reported active ingredients of AR were retrieved by searching the ETCM and TCMIP databases. In this study, we mainly focused on the active flavonoid compounds, and in line with the OB ≥ 30% and DL ≥ 0.18 thresholds, five active flavonoids were selected: For, Iso, Cal, Que, and Kae (Figure 2A; Supplementary Table S1). These five flavonoids were used for the subsequent network pharmacology analysis and experimental validation.

### Target Prediction and Analysis

**FIGURE 3:**
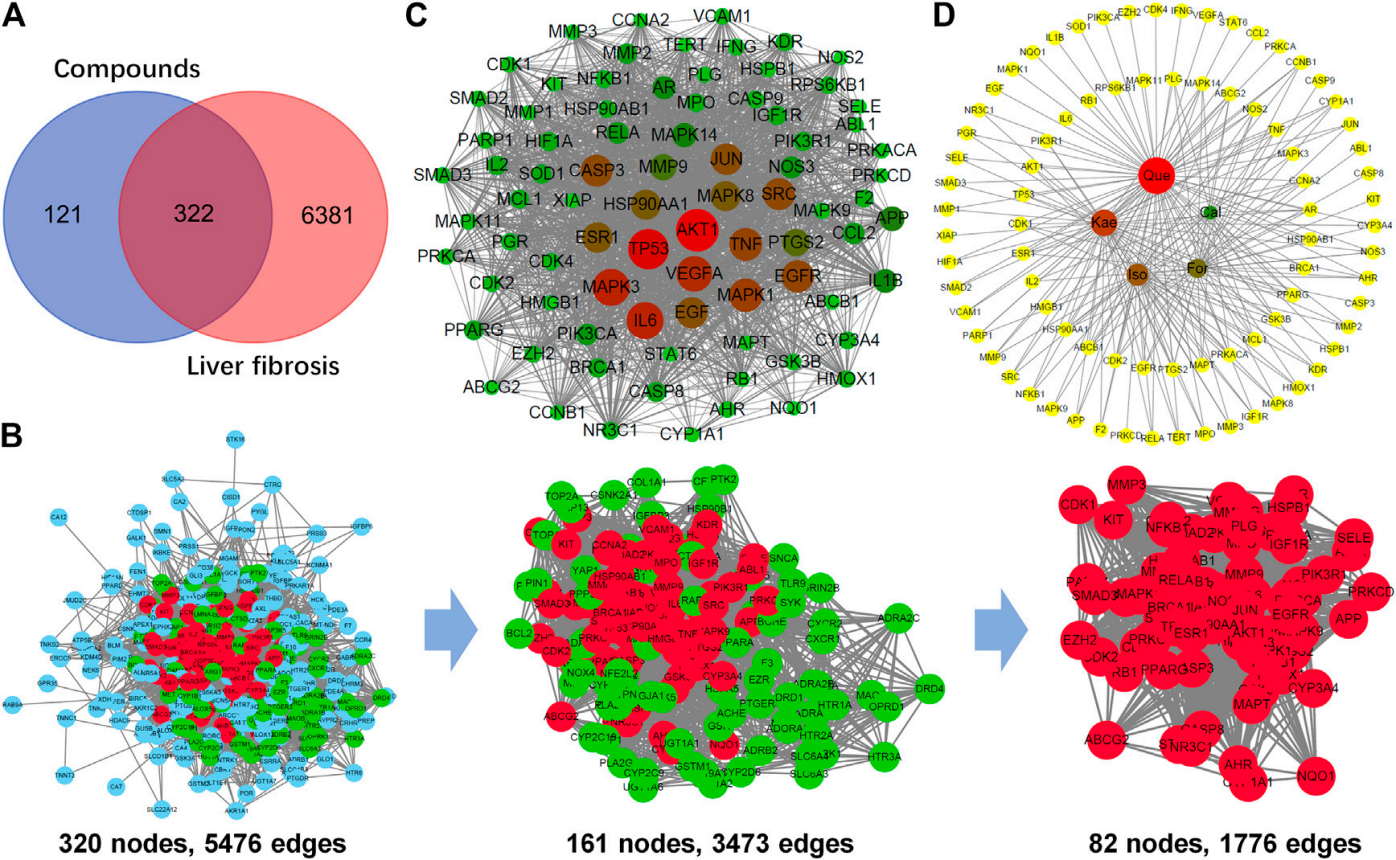
Analysis of the key targets of the flavonoids for the treatment of liver fibrosis. **(A)** Venn diagram summarizing the differential targets of the flavonoids and liver fibrosis. **(B)** The process of topological screening for the protein-protein interaction (PPI) network. Finally, 82 candidate targets were predicted. **(C)** The PPI network of the 82 nodes. Different colors represent nodes of different sizes. The red nodes represent the large hub nodes and the green nodes represent the small hub nodes. The node size is proportional to the target degree in the network. **(D)** Compound target network (C-T network) of the flavonoids and 82 key targets.

In this process, we conducted target fishing on the five flavonoids based on the TCMSP, PubChem, STITCH, SuperPred and Swiss Target Prediction databases, obtaining 443 related targets, among which there were 141 for Cal, 144 for Iso, 167 for For, 212 for Kae and 315 for Que (Figure 2B). All of these targets were combined and identified using the UniProt database. By searching the GeneCards and DisGeNET databases for related research reports, 6,703 targets were obtained, which are closely related to the occurrence and development of “HF” or “liver fibrosis.” Three hundred and twenty-two targets that matched the related targets of the five flavonoids and HF were then collected as the potential targets of the five flavonoids against HF (Figure 3A). Then, the PPI network of those 322 targets was established in the database String. There were 320 nodes and 5,476 edges in total.

Afterward, the topological feature analysis of the PPI network was conducted with the Cytoscape software platform based on three major parameters: degree, betweenness, and closeness. Those targets greater than the median were chosen to be key targets, then, hub nodes were established from those five anti-HF flavonoids. In the initial screening, the thresholds were set at degree ≥ 28, betweenness ≥ 0.00115124, and closeness ≥ 0.47399703. At last, 3,473 edges and 161 hub nodes were screened. Thereafter, those 161 key targets were subjected to secondary screening, at the thresholds of degree ≥ 43, betweenness ≥ 0.00312659, and closeness ≥ 0.51203852. The second screening ended with 82 large hub nodes and 1,776 edges (Figure 3B). Then, Cytoscape was adopted for visualizing and integrating those PPI network-involved topological parameters for those 82 screened key targets. The crucial biotargets were obtained and visualized, which included AKT1, TP53, MAPK3, IL6, TNF, JUN, MAPK1, and EGFR, etc (Figure 3C). Finally, based on 82 key targets, a large hub node-compound network was further constructed according to the five flavonoids. It was observed that Cal held relevancy to 23 key targets, For was related to 34 key targets, Iso was connected to 41 key targets, Kae was related to 50 key targets, whereas Que was associated with 72 key targets (Figure 3D).

### GO Biological Process and KEGG Pathway Enrichment Analysis

**FIGURE 4:**
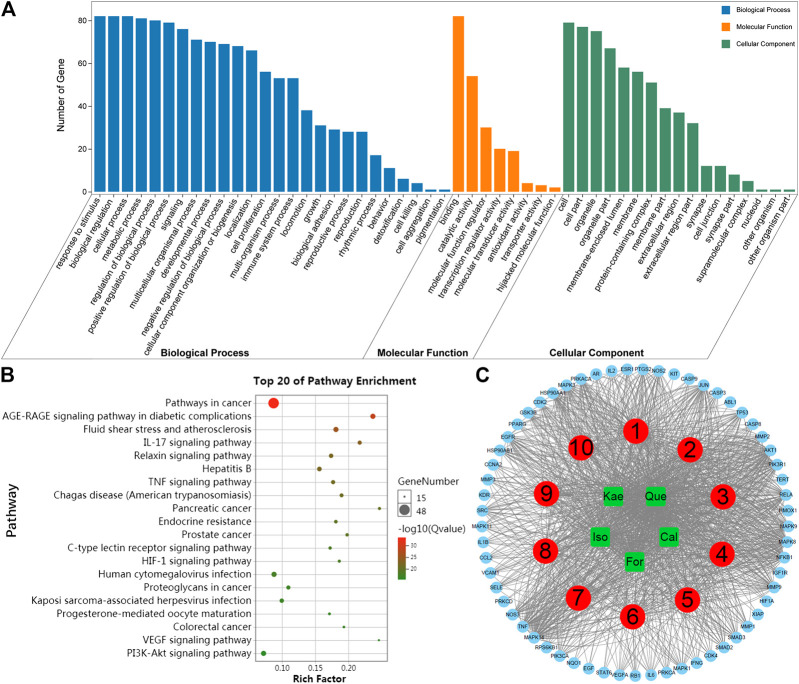
Enrichment analysis of the key targets of the flavonoids against liver fibrosis. **(A)** GO enrichment analysis of the 82 key targets. Biological processes, cellular components, and molecular function terms were performed on the targets. The top significant (the cutoff criterion for a significant difference was *p* < 0.05) GO categories are shown. **(B)** KEGG enrichment analysis of the 82 key targets. The top 20 KEGG pathway enrichments are displayed, and only terms with FDR < 0.05 were selected for analysis. **(C)** Compound-target-pathway network. The red round nodes represent the top ten pathways, the green round nodes represent the flavonoids, and the blue triangles represent the related targets.

The top significant (cutoff criterion with a significant difference of *p* < 0.05) GO categories are shown in Figure 4A. In biological processes (BPs), the common targets were mainly enriched in cellular response to chemical stimuli (GO: 0070887), response to oxygen-containing compounds (GO: 1901700), response to organic substances (GO: 0010033), cellular response to oxygen-containing compounds (GO: 1901701), cellular response to organic substances (GO: 0071310), etc. In molecular functions (MFs), the common targets were mainly associated with enzyme binding (GO: 0019899), identical protein binding (GO: 0042802), protein kinase activity (GO: 0004672), kinase binding (GO: 0019900), and protein kinase binding (GO: 0019901), etc. In addition, cellular component (CC) analysis showed that the common targets were the membrane-enclosed lumen (GO: 0031974), organelle lumen (GO: 0043233), intracellular lumen (GO: 0070013), nucleoplasm (GO: 0005654), and cytoplasmic part (GO: 0044444), etc.

To further reveal the potential mechanism of the five flavonoids against HF, KEGG analysis was carried out on those 82 key targets. The top 20 KEGG pathway enrichments were displayed based on the threshold of FDR < 0.01; for instance, AGE-RAGE signal transduction pathway in diabetes-related complications (ko04933), pathways in cancer (ko05200), fluid shear stress and atherosclerosis (ko05418), pancreatic cancer (ko05212), IL-17 signal transduction pathway (ko04657), hepatitis B (ko05161), relaxin signal transduction pathway (ko04926), TNF signaling pathway (ko04668), Chagas disease (American trypanosomiasis) (ko05142), and endocrine resistance (ko01522), etc (Figure 4B). Moreover, those 10 most significant KEGG pathways were used to construct the compound-target-pathway network (Figure 4C). The characteristics of multiple components, multiple targets and multiple pathways of the active ingredients against liver fibrosis were revealed through the construction of a network. Further analysis revealed that the molecular mechanisms were mainly concentrated in immune and inflammatory pathways, with the main pathway being the classical IL-17 signaling pathway (Supplementary Figure S1). The key targets of the IL-17 signaling pathway mainly include NF-κB, AP-1, IL-6, IL-1β, TNFα, IFN-γ, CCL2, COX2, MMP1, MMP3, and MMP9.

### The Five Flavonoids of AR Did Not Show Cytotoxicity to HSCs

**FIGURE 5:**
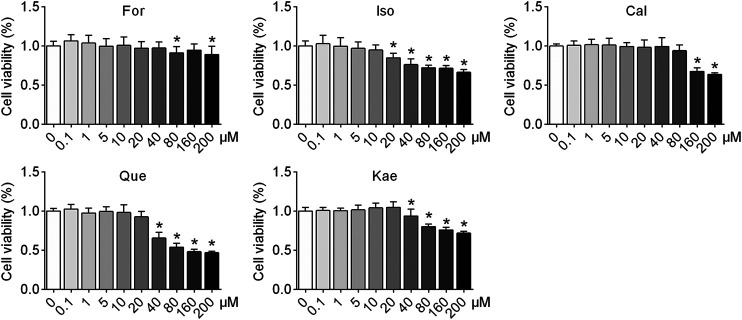
The five flavonoids of AR had no cellular toxicity in HSCs at concentrations of 1, 5, and 10 μM. HSC-T6 cells were treated with For, Iso, Cal, Que, and Kae at concentrations ranging from 0.1 to 200 μM for 24 h. Cell viability was detected by performing a CCK-8 assay (*n* = 3). Data are expressed as the mean ± SD. *p < 0.05 vs. the blank control.

To elucidate the efficacy of the five flavonoids against HF, the first step should be to investigate the cellular toxicity of these compounds against HSCs. The results showed that in resting cells, a concentration of 20 μM Iso produced a significant effect on cell viability in HSC-T6 cells (*p* < 0.05), whereas 0.1–10 μM flavonoids had no toxicity. Thus, the five flavonoids at concentrations of 1, 5, and 10 μM were chosen for the following experiments, excluding the possibility that these five flavonoids inhibited HSC activation *via* cytotoxicity (Figure 5).

### The Five Flavonoids Were Able to Suppress HSC Activation

**FIGURE 6:**
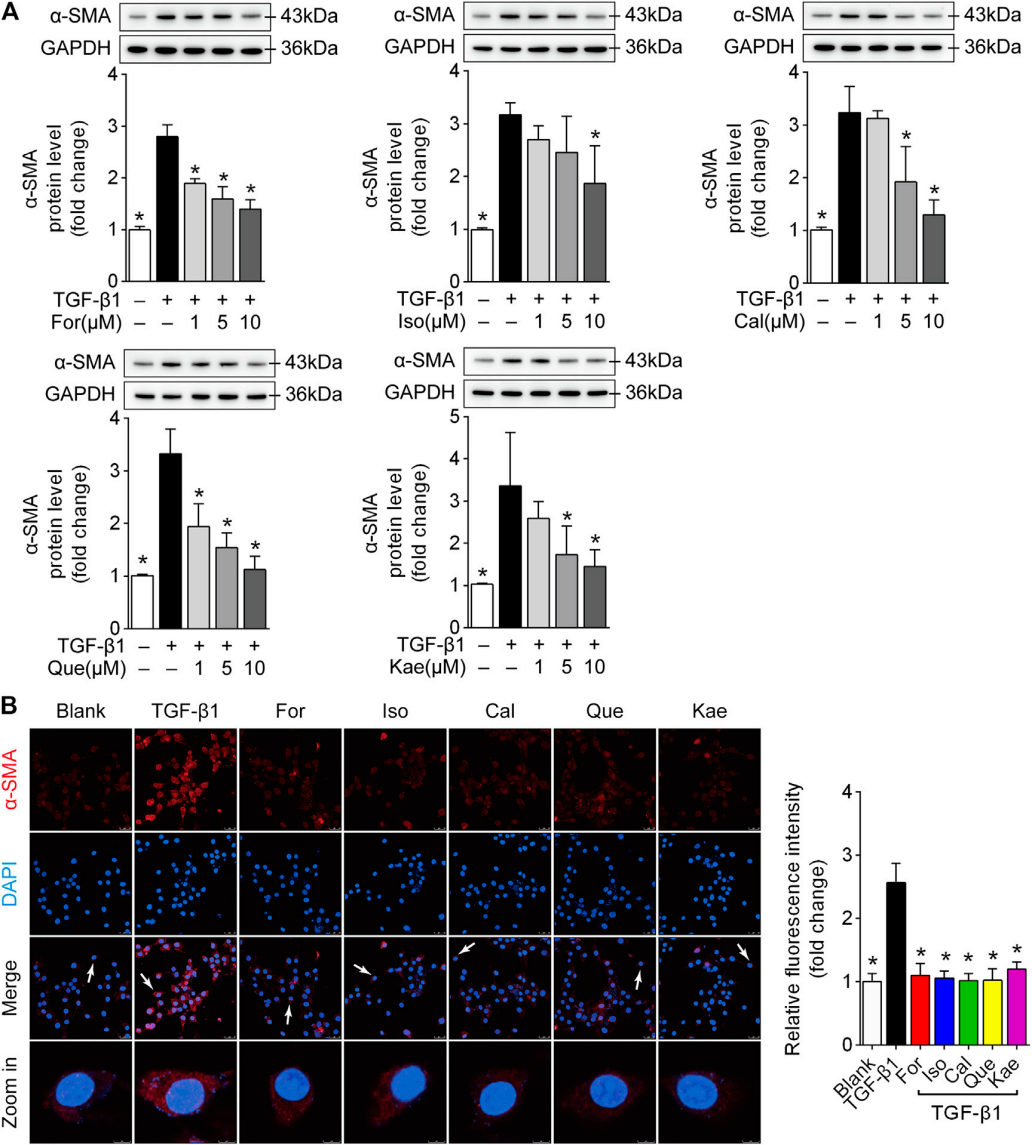
The five flavonoids were able to suppress HSC activation. HSC-T6 cells were stimulated with TGF-β1 (10 ng/ml) for 24 h and prepared for further experiments. **(A)** Alpha-SMA protein expression in TGF-β1-induced HSC-T6 cells (*n* = 3). **(B)** Confocal scanning images and quantified data of α-SMA in HSC-T6 cells stimulated with TGF-β1 (10 ng/ml) for 24 h (*n* = 3), scale bar = 50 μm for 400×. Data are expressed as the mean ± SD. *p < 0.05 vs. TGF-β1 treatment.

In order to further study the effects of the five flavonoids on HSC transdifferentiation, the expression of α-SMA, a hallmark in the process of HSC activation (31258636), was detected in TGF-β1-challenged HSC-T6 cells. As suggested by Western blotting, those five flavonoids significantly decreased α-SMA protein level within HSC-T6 cells induced by TGF-β1 (Figure 6A; *p* < 0.05). Additionally, visualization by confocal microscopy and the quantified data confirmed the functional role of the five flavonoids in reducing α-SMA overexpression induced by TGF-β1 (Figure 6B; *p* < 0.05). These data revealed that the five flavonoids of AR impeded HSC activation, possibly contributing to the prevention of liver fibrosis.

### The Five Flavonoids Regulated the Phenotypes of HF

**FIGURE 7:**
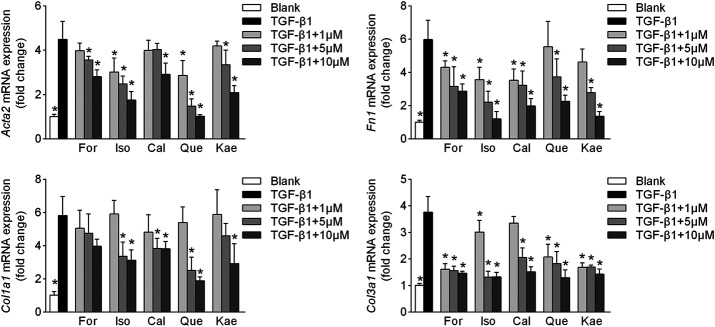
HF phenotypes were improved by the five flavonoids of AR. HSCs were stimulated with TGF-β1 (10 ng/ml) for 24 h and prepared for RT-qPCR experiments. Illustrations are shown for the HF phenotypes of *Acta2* (upper left panel, *n* = 4), *Fn1* (upper right panel, *n* = 4), *Col1a1* (lower left panel, *n* = 4), and *Col3a1* (lower right panel, *n* = 4). Data are expressed as the mean ± SD. *p < 0.05 vs. TGF-β1 treatment.

In the process of HSC transdifferentiation into myofibroblasts, increasing amounts of α-SMA, collagen, fibronectin, and other ECM proteins induced by TGF-β1 eventually give rise to HF (Parola and Pinzani, 2019). The results showed that TGF-β1 stimulation remarkedly increased *Acta2*, *Fn1*, *Col1a1* and *Col3a1* mRNA expression approximately 4∼6-fold (*p* < 0.001) in HSC-T6 cells, whereas these effects were significantly reversed by For in a dose-dependent manner (Figure 7; *p* < 0.05). Similar results were noted for other flavonoid treatments (*p* < 0.05). These results demonstrated that the five flavonoid interventions increased the resistance of liver stellate cells to TGF-β1-induced fibrogenic responses.

### The Inflammatory Pathway Might Be Involved in the Antifibrotic Effects of the Five Flavonoids

**FIGURE 8:**
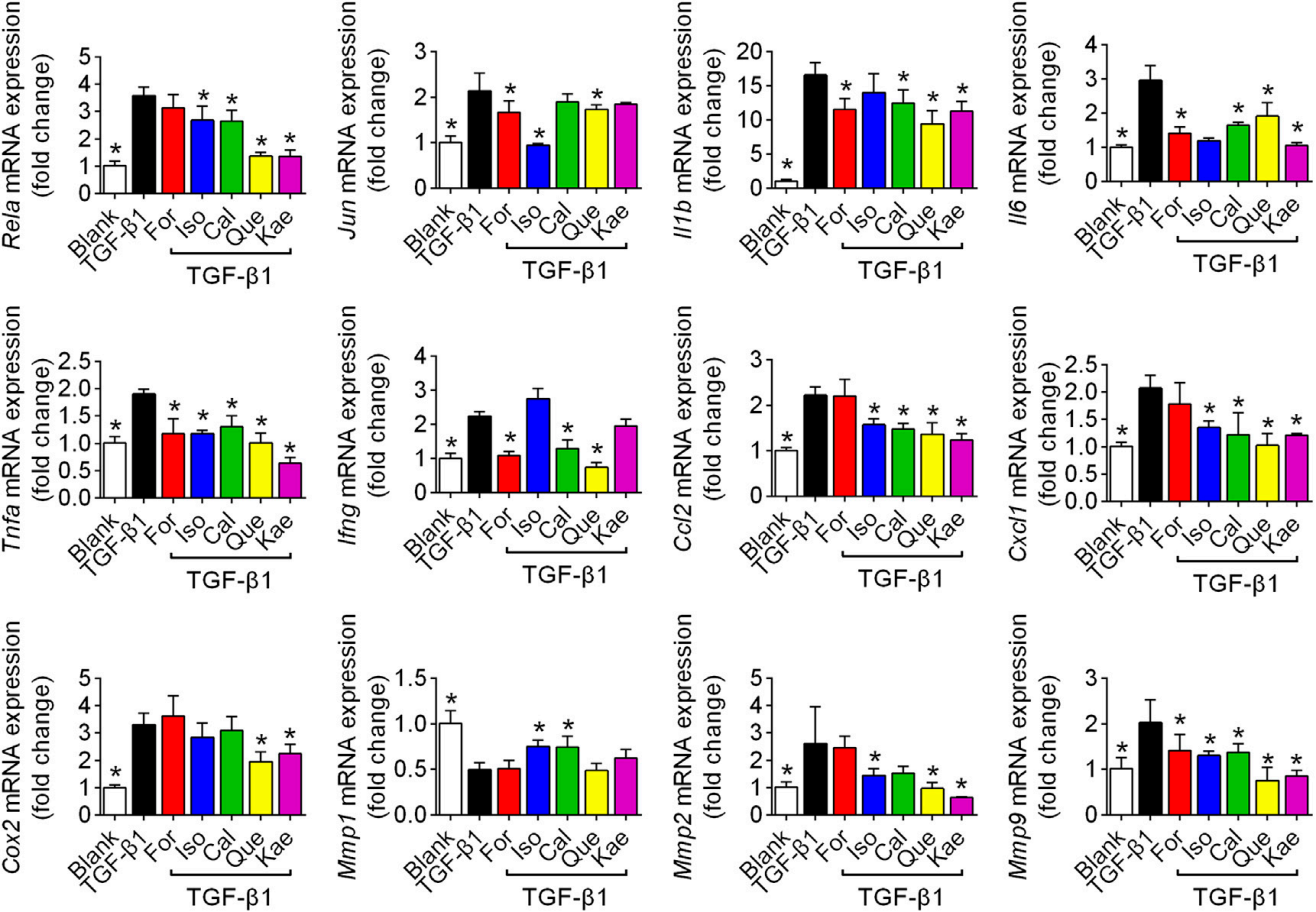
Inflammatory factors were involved in the process of flavonoid inhibition of HSC activation. HSCs were stimulated with TGF-β1 (10 ng/ml) for 24 h and prepared for RT-qPCR experiments. The figures show the mRNA expression of different inflammatory factors with or without interference with For, Iso, Cal, Que, or Kae in TGF-β1-induced HSC-T6 cells (*n* = 4). Data includes *Rela*, *Jun*, *Il1b*, *Il6*, *Tnfa*, *Ifng*, *Ccl2*, *Cxcl1*, *Cox2*, *Mmp1*, *Mmp2*, and *Mmp9*. Data are expressed as the mean ± SD. *p < 0.05 vs. TGF-β1 treatment.

Based on the enrichment analysis by using network pharmacology, we noted a number of differentially expressed genes and proteins associated with the inflammatory process, including the inflammatory cytokines IL-1β, IL-6, tumor necrosis factor alpha (TNF-α), and interferon gamma (IFNγ); chemokines CCL2 and CXCL1; the enzyme COX2; and matrix metalloproteinases (MMPs) MMP1, MMP2, and MMP9. We detected these factors by utilizing the RT-qPCR method. The results showed that TGF-β1 stimulation increased the mRNA expression of *Rela*, *Jun*, cytokines *Il1b*, *Il6*, *Tnfa*, and *Ifng*, chemokines *Ccl2*, and *Cxcl1*, the related enzyme *Cox2*, and MMP family members *Mmp2*, and *Mmp9* from ∼2 to ∼17-fold (*p* < 0.05), whereas the five flavonoids could effectively reverse these effects in HSC-T6 cells (*p* < 0.05). The protein MMP1 was able to degrade ECM in HSCs, especially type I and type III collagen. As Figure 8 shows, the five flavonoids could effectively elevate *Mmp1* expression (*p* < 0.05); however, there was no significant difference after treatment with For, Que, or Kae. Collectively, these compounds were shown to significantly suppress the inflammatory process (Figure 8).

### The Five Flavonoids of AR Exhibited Anti-Inflammatory Effects *via* the NF-κB Signaling Pathway

**FIGURE 9:**
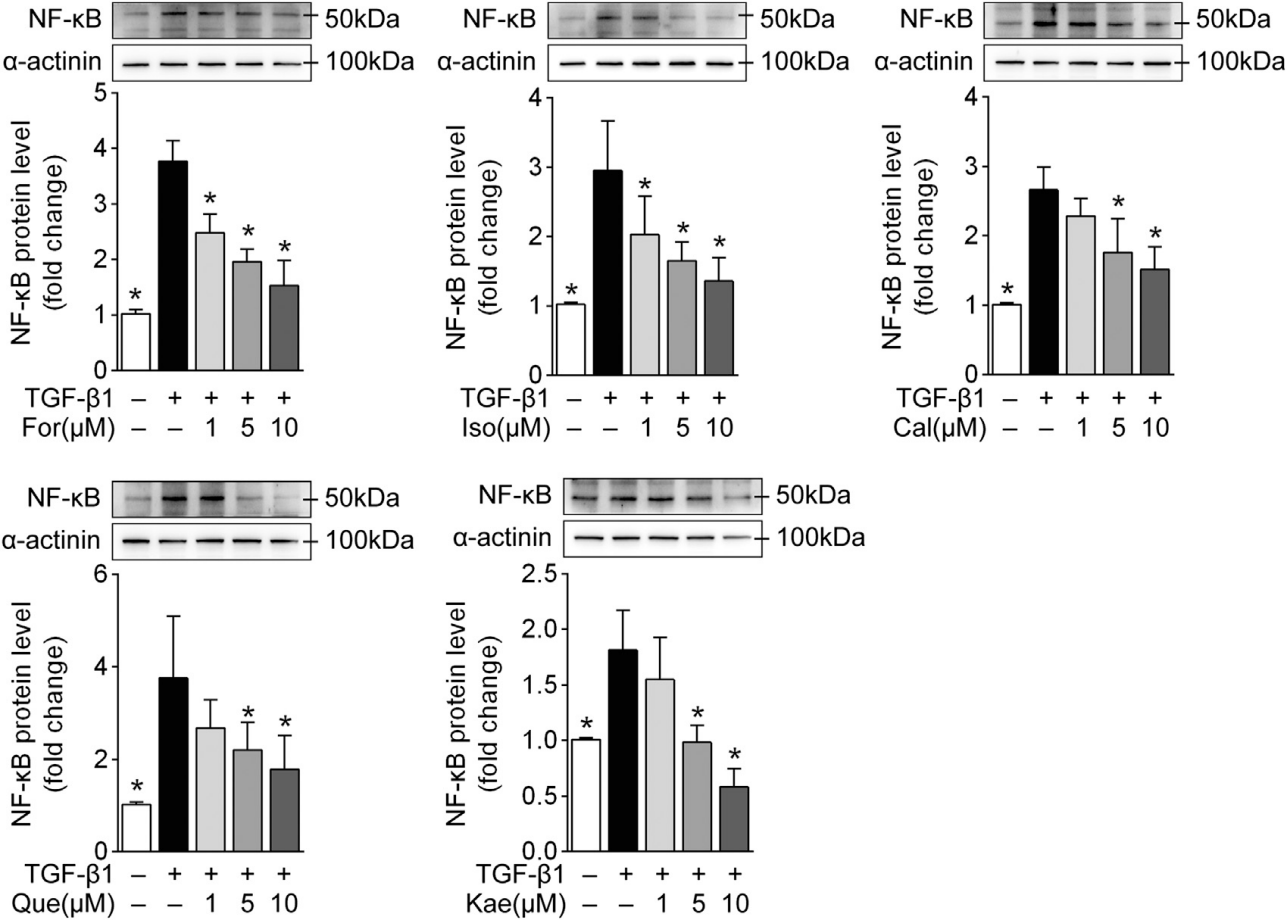
The five flavonoids might exhibit antifibrotic effects *via* the NF-κB signaling pathway. HSC-T6 cells were stimulated with TGF-β1 (10 ng/ml) for 24 h and prepared for protein level analysis. The diagrams show the NF-κB protein level with or without interference with For, Iso, Cal, Que, and Kae in TGF-β1-induced HSC-T6 cells (*n* = 3). Data are expressed as the mean ± SD. *p < 0.05 vs. TGF-β1 treatment.

The results from KEGG enrichment showed that the IL-17 pathway, mainly including NF-κB, AP-1 and various related inflammatory factors, might be involved in the treatment of the five flavonoids against liver cirrhosis. Accordingly, it needs to be confirmed whether these flavonoids exhibit antifibrotic effects *via* the regulation of NF-κB or AP-1. Although the five flavonoids showed regulation in AP-1 at the transcriptional level to a certain extent (Figure 8), the protein level had no marked change. Moreover, it was found that there were higher protein levels of NF-κB after TGF-β1 treatment than in the flavonoid treatment groups. Moreover, Western blot analysis and the semiquantitative illustration demonstrated that all five flavonoids could decrease NF-κB protein levels in a dose-dependent manner (Figure 9; *p* < 0.05). These data suggested that the anti-inflammatory effects of the flavonoids involved NF-κB, indicating that these active flavonoids repressed HSC activation *via* the NF-κB signaling pathway.

### Analysis of Molecular Docking Results

**FIGURE 10:**
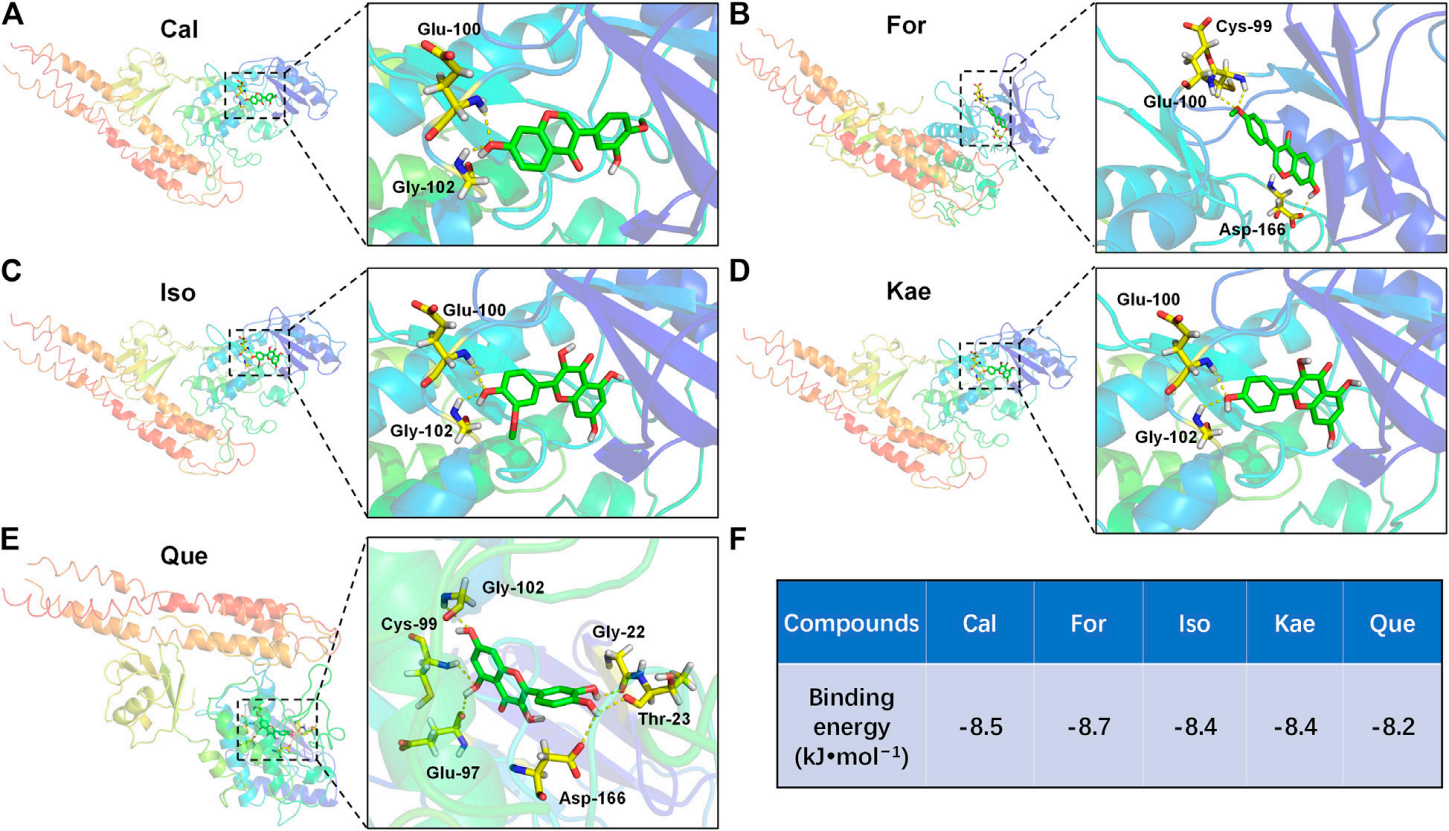
Molecular docking diagrams of IKKβ with **(A)** Cal, **(B)** For **(C)** Iso, **(D)** Kae, and **(E)** Que. **(F)** The binding energies for the five flavonoids docked into the IKKβ crystal structure. The yellow dashed lines show H-bonds.

The docking results of the five flavonoids with the target protein IKKβ are shown in Figure 10. The residues docked with the molecular ligands are shown as yellow sticks. Amino acid residues Glu100 and Gly102 in the crystal structure of IKKβ formed hydrogen bonds with Cal (Figure 10A). For could bind to the residues Cys99, Glu100, and Asp166 in the IKKβ crystal structure by hydrogen bonds (Figure 10B). Both Iso and Kae could bind to the residues Glu100 and Gly102 of the IKKβ crystal structure by hydrogen bonds (Figures 10C,D). Amino acid residues Gly22, Thr23, Glu97, Cys99, and Gly102 in the crystal structure of IKKβ formed hydrogen bonds with Que (Figure 10E). The docking scores for the five flavonoids (Cal, For, Iso, Kae, and Que) with the IKKβ crystal structure were −8.5, −8.7, −8.4, −8.4 and −8.2 kcal/mol, respectively (Figure 10F). The docking score represents the binding affinity, and when the score is lower, the binding affinity is stronger. An affinity <−7 indicates strong binding activity (19,499,576). Our results indicate that the flavonoids have a strong binding affinity for IKKβ.

## Discussion

Liver cirrhosis severely decreases the quality of life of patients. It is of great significance to seek an effective therapeutic strategy to restrain liver disease deterioration. AR, a well-known traditional Chinese medicine, has been reported to be therapeutically effective against liver cirrhosis. However, the molecular mechanism has yet to be completely deciphered. Flavonoids are the main bioactive compounds involved in the functions of AR. Thus, in the current study, investigation into the effects and mechanism of the flavonoids from AR against liver fibrosis was conducted based on a network pharmacology approach coupled with experimental validation and molecular docking analysis. These results expand our knowledge pertaining to the basis of the effects and mechanism of AR-induced anti-liver fibrosis.

The use of TCMs, including AR, in the prevention and treatment of liver fibrosis has increased worldwide due to their privileged properties. However, it is difficult to elucidate the effects of AR because of its multiple compounds, multiple targets, multiple pathways, and multiple mechanisms of action (Guo et al., 2019), which greatly restrict the processes of modernization and internationalization of AR. In recent years, along with the generalization of systems biology, network pharmacology, which integrates chemoinformatics, bioinformatics, network biology, network analysis and traditional pharmacology, has emerged as a powerful tool to analyze the mechanism of the complex components of TCMs (Berger and Iyengar, 2009; Li and Zhang, 2013; Hao and Xiao, 2014). Thus, this study is the first to comprehensively screen the active ingredients of AR, predict the targets of these active ingredients and the genes related to liver fibrosis and analyze the potential mechanism of AR against liver fibrosis by using a network pharmacology approach.

After screening AR for OB ≥ 30% and DL ≥ 0.18, a total of 87 reported active ingredients of AR were retrieved by searching the TCMSP database. Modern pharmacological studies indicate that flavonoids are the leading favorable compounds related to different AR pharmacological effects and efficacy (Guo et al., 2019). However, whether the flavonoids of AR can exert inhibitory effects on HF is not yet completely understood. There is little information regarding how flavonoids contribute to the treatment of HF by AR. There is an urgent need for systematic evaluation of the flavonoid components in AR to determine the definite bioactive component responsible for the activity of AR against HF. Thus, five potential bioactive flavonoids were selected: For, Iso, Cal, Que, and Kae (Figure 2A; Supplementary Table S1), for subsequent network pharmacology analysis and experimental validation. Then, the PPI network of candidate targets for flavonoids in the treatment of liver fibrosis was established based on the flavonoid and liver fibrosis target networks with 322 overlapping genes (Figures 2B,3A).

Afterward, the topological feature analysis of the PPI network was conducted according those three main parameters, namely, degree, closeness and betweenness. A total of 82 targets greater than the median were screened to be the key targets for those five anti-HF flavonoids (Figures 3B,C). It was apparent that most of these targets, including AKT1, TP53, MAPK3, IL6, TNF, JUN, MAPK1, EGFR, and many other inflammation-related proteins, were closely involved in the inflammation process. It was also observed that Que was linked to the greatest number of key targets, followed by Kae, Iso, For, and Cal (Figure 3D), suggesting that Que could be the most significant compound in AR against liver fibrosis.

Next, crucial target-related enrichment analyses of the GO and KEGG pathways were performed. GO enrichment analysis, which was based on three different terms, including BP, CC, and MF terms, was used to identify the biological mechanisms of key targets in disease (Ashburner et al., 2000). Based on the GO terms, it was proposed that the pharmacological effects of AR against liver fibrosis occurred by simultaneously activating various biological processes, cellular components, and molecular functions (Figure 4A). KEGG database can be adopted to identify potential target biological relevance and systematic functions (Chen et al., 2015). After KEGG enrichment analysis, it was found that the five flavonoids could potentially suppress liver fibrosis through multiple pathways, including pathways in cancer, the AGE-RAGE signal transduction pathway in diabetes-related complications, fluid shear stress and atherosclerosis, the IL-17 signal transduction pathway, the relaxin signal transduction pathway, hepatitis B, and the TNF signaling pathway, etc (Figures 4B,C). Further analysis revealed that the molecular mechanisms were mainly concentrated in the immune and inflammatory pathways, with the main pathway being the classical IL-17 signaling pathway (Supplementary Figure S1). The key targets of the IL-17 signaling pathway include NF-κB, AP-1, IL-6, IL-1β, TNFα, IFN-γ, CCL2, COX2, MMP1, MMP3, and MMP9, which all exert vital parts in HF development (Tan et al., 2020; Zhou et al., 2020). Accordingly, in addition to the network pharmacology analysis, we next performed experimental validation to clarify the potential inflammatory mechanism of the flavonoids against liver fibrosis.

HSCs are pericytes found in the perisinusoidal space of the liver. Stellate cells are the major cell type involved in liver fibrosis, which is the formation of scar tissue in response to liver damage (Tsuchida and Friedman, 2017). Based on this, we used HSCs for subsequent biological verification experiments. To determine the effects of the five flavonoid compounds screened by network pharmacology on HSCs, the first step was to explore whether these compounds had cellular toxicity. Figure 5 shows that except for the Que panel, the cell survival rate of all treatments was higher than 70% in the concentration range of 0.1–200 μM. The cell viability was greater than 70% in the 40 μM group of HSCs treated with Que. However, in all flavonoid intervention groups, a concentration less than or equal to 10 μM compound did not decrease the survival rate of HSCs, indicative of the nontoxicity of the five flavonoids for further experiments.

Liver fibrogenic responses are characterized by the differentiation of HSCs into myofibroblasts, and excessive ECM deposition is a major consequence of myofibroblast activation. HSCs activation to the proliferating fibrogenic myofibroblasts has been extensively recognized to be a pivotal factor of HF in human or experimental liver damage (Parola and Pinzani, 2019). Myofibroblasts are important but transient mediators of normal wound contraction and are characterized phenotypically by their high levels of α-SMA. Therefore, α-SMA is often used as a marker of myofibroblast formation (Peng et al., 2019). In the present study, we applied multiple biological methods for evaluating those flavonoid effects on α-SMA expression within the HSC-T6 cells treated with TGF-β1. As indicated, the five flavonoids had a remarkable inhibitory effect on the transdifferentiation of HSCs (Figure 6). Upon HSC activation, formative myofibroblasts secrete large amounts of ECM. Once secreted, the formative myofibroblasts then aggregate with the existing matrix, which eventually leads to liver fibrosis. Collagens are the most abundant protein in the ECM, especially type I collagen (Col-I) and type III collagen (Col-III). Collagen fibers are connected to cells by fibronectins in the ECM (Ricard-Blum, 2011). Hence, these fibrogenic factors are good indicators of the severity of HF. In Figure 7, we detected all of these related HF phenotypes, and the RT-qPCR analysis showed that the main flavonoids of AR could counteract the effects induced by TGF-β1 in HSC-T6 cells. Given the critical role of α-SMA and ECM deposition in HSC activation and differentiation, these results indicated that the main flavonoids from AR restrained HF by inhibiting HSC activation, an effect likely resulting from interference with the inflammatory process when the KEGG map was taken into account.

Inflammatory pathways are associated with numerous diseases. The scientific evidence and KEGG results from our work suggested that the IL-17 pathway, mainly NF-κB and its associated inflammatory factors, accounts for an evolutionarily conservative pathway that can regulate HF (Luedde and Schwabe, 2011). NF-κB is suggested to regulate the liver fibrogenesis mainly in three diverse cell fractions, including regulating hepatocyte injury, inflammatory signals, and fibrogenic responses of HSCs. Among these, there is growing evidence that NF-κB acts as a key mediator of fibrosis in the transdifferentiation of HSCs to myofibroblasts (Friedman, 2004). The potential mechanisms appear through direct fibrogenic effects, anti-apoptotic effects, and secretion of macrophage-recruiting chemokines. NF-κB participates in down-regulating members of the miR-29 family within HSCs, thereby increasing the levels of all collagens together with additional ECM proteins within HSCs (Roderburg et al., 2011). Since NF-κB is primarily composed of the p50 and p65 subunits, we investigated different subunits at different biological levels. In this study, we confirmed that there was a remarkable elevation in NF-κB mRNA (*Rela*) and protein (p50) levels in the process of HSC activation, and all five flavonoids reversed these effects in response to TGF-β1 stimulation in HSC-T6 cells. As shown in Figures 8, 9, Que demonstrated the optimal effect among the five flavonoids.

Activation of NF-κB also promotes the secretion of various inflammatory mediators. Indeed, in the network pharmacology study, we identified several related inflammatory factors, including cytokines, chemokines, enzymes, and the MMP family. Cytokines, the small proteins with the molecular weight of 5–20 kDa, play vital roles in cell signaling. Of these cytokines, IL-1β can directly trigger the synthesis of collagen together with the proliferation of myofibroblasts (Kähäri et al., 1987). IL-6, IFN-γ, and TNF-α are secreted by T cells, biliary cells, and Kupffer cells, respectively, which contributes to HSC activation (Bataller and Brenner, 2005). As Figure 8 shows, Que and Kae might have better inhibitory effects on these cytokines than the other flavonoids. In addition to cytokines, the increased secretion of chemokines such as CCL2 and CXCL1 results in inflammatory cell influx, like the macrophages, and they show interactions with HSCs and have certain effect on HSC activation as well as wound healing (Seki et al., 2007). In this study, we found that all five flavonoids could downregulate chemokine expression induced by TGF-β1.

Furthermore, we analyzed a vital inducible sensor, COX-2, which exerts a vital part in liver fibrogenesis development. COX-2 is a rate-limiting enzyme responsible for catalyzing arachidonic acid in converting to thromboxane and inflammatory prostaglandins (Yang et al., 2020). Despite the controversial relationship between COX-2 and TGF-β1, we observed a marked increase in *Cox2* mRNA expression after TGF-β1 induction, and this elevation was reversed after flavonoid treatment. Accompanying cellular activation, ECM constituents change from the matrix with abundant laminin and collagen IV to the concentrated interstitial ECM, which suggested the possible occurrence of proteolytic degradation for altering sinusoid microenvironment and HSCs fate (Han, 2006). As a matter of fact, members in MMP family are rapidly expressed via HSCs to respond to a variety of hepatic toxins. Depending on timing and other reasons, MMPs play dual roles in liver fibrosis. It has been reported that MMP2 and MMP9 are expressed in human fibrotic injury, while MMP1 can degrade ECM in HF (Takahara et al., 1997; Iimuro et al., 2003). Our work not only confirmed this phenomenon but also noted that Kae showed better MMP family regulatory ability than the other flavonoids. However, MMPs have different functions during the different processes of HF, and more in-depth experiments are needed. Besides, the antifibrotic effects of the five flavonoids should be further demonstrated using pathway inhibitors *in vitro* and animal model *in vivo*. Transgenic animal experiments have been conducted to clarify the exact mechanism(s).

Finally, the potential mechanism of AR flavonoids against liver fibrosis was further validated by using molecular docking. Molecular docking is a quick and effective method of predicting the binding affinity between TCM ingredients and their targets based on the spatial structure of ligands and receptors (Gan et al., 2019). Molecular docking analysis was conducted by using AutoDock Vina software, which may serve as the AutoDock successor docking analysis, due to its effect on apparently improving performance and accuracy in comparison with those of Lamarckian Genetic Algorithm (Kist et al., 2018). Based on results of network pharmacology, it was known that flavonoids can possibly suppress liver fibrosis by inhibiting the IL-17 signaling pathway, which includes NF-κB, AP-1, IL-6, IL-1β, TNFα, IFN-γ, CCL2, COX2, MMP1, MMP3, and MMP9. The experimental validation results further confirmed that the flavonoids could exhibit potent inhibitory activity against liver fibrosis *via* suppression of NF-κB and its various downstream inflammatory factors. NF-κB represents the key transcription factor related to the inflammatory signaling cascade (Hoesel and Schmid, 2013). Normally, NF-κB can be found in cytoplasm, which can be inactivated by the IκB inhibitors. The NF-κB inflammatory activation is dependent on the IKKβ-mediated IκB protein phosphorylation, as well as p50–p65 subunit nuclear translocation in NF-κB, thus inducing the expression of proinflammatory cytokines (Hoesel and Schmid, 2013). As a result, specifically inhibiting IKKβ is a reasonable way to more effectively treat inflammatory disorders, like HF (Baig et al., 2018). The results of molecular docking showed that the flavonoids had high affinity for IKKβ (Figure 10), suggesting that the mechanism of the flavonoids against liver fibrosis may be related to suppression of the NF-κB pathway through effective inhibition of IKKβ.

## Conclusion

This study aimed to undertake network pharmacology analysis coupled with experimental validation and molecular docking to investigate the effects and mechanism of AR flavonoids against liver fibrosis. The network pharmacology analysis findings showed that the flavonoids from AR exerted their pharmacological effects against liver fibrosis by modulating multiple targets and pathways. The experimental validation results suggested that flavonoids might play an anti-inflammatory role in the treatment of liver fibrosis by mediating inflammation signaling pathways. Our molecular docking study results also indicated that the mechanism of the flavonoids against liver fibrosis may be related to suppression of the NF-κB pathway through effective inhibition of IKKβ. These findings not only provide a scientific basis for clarifying the mechanism of action of AR in the treatment of liver fibrosis but also suggest a novel promising therapeutic strategy for the treatment of liver fibrosis. However, as this study was based on data mining and experimental validation, further clinical validation studies should be undertaken to determine the role of AR in liver fibrosis.

## Data Availability Statement

The original contributions presented in the study are included in the article/Supplementary Material, further inquiries can be directed to the corresponding authors.

## Author Contributions

JW and LA designed the experiments and wrote the manuscript. LA, YFL, MK, LL and YML carried out the experiments and analyzed the data. JW and ZL supervised and corrected the manuscript.

## Funding

This work was supported by grants from the National Natural Science Foundation of China (81703803, 81930114), the Natural Science Foundation of Guangdong Province (2017A030310464), the Project of Guangzhou University of Chinese Medicine (QNYC20190103), the Basic and Applied Basic Research Fund Project of Guangdong Province (2019A1515110202), the China Postdoctoral Science Foundation (2020M672604), Guangdong Key Laboratory for translational Cancer research of Chinese Medicine (2018B030322011), and Key-Area Research and Development Program of Guangdong Province (No. 2020B1111100004).

## Conflict of Interest

The authors declare that the research was conducted in the absence of any commercial or financial relationships that could be construed as a potential conflict of interest

## Glossary

*Acta2*: alpha smooth muscle actin
α-SMA: alpha smooth muscle actin
AR: *Astragalus membranaceus* (Fisch.) Bunge
BP: biological process
Cal: calycosin
CC: cellular component
*Ccl2*: C-C motif chemokine two
Col-I: type I collagen
Col-III: type III collagen
*Col1a1*: type I collagen
*Col3a1*: type III collagen
*Cox2*: prostaglandin G/H synthase two
*Cxcl1*: growth-regulated alpha protein
DL: drug similarity
ECM: extracellular matrix
*Fn1*: fibronectin
For: formononetin
*Gapdh*: glyceraldehyde-3-phosphate dehydrogenase
GO: gene ontology
HF: hepatic fibrosis
HSCs: Hepatic stellate cells
*Ifng*: interferon gamma
*Il1β*: interleukin-1 beta
*Il6*: interleukin-6
Iso: isorhamnetin
*Jun*: transcription factor AP-1
Kae: kaempferol
KEGG: encyclopedia of genes and genomes
MF: molecular function
*Mmp1*: matrix metalloproteinase one
*Mmp2*: matrix metalloproteinase two
*Mmp9*: matrix metalloproteinase nine
NF-κB: nuclear factor kappa B
OB: oral bioavailability
PPI: protein to protein interaction
Que: quercetin
*Rela*: nuclear factor kappa B subunit p65
TCMs: traditional Chinese medicines
TCMSP: traditional Chinese medicine systems pharmacology
TGF-β1: transforming growth factor beta one
*Tnfa*: tumor necrosis factor
