# Supplementary material for: Integrating Network Pharmacology and Experimental Validation to Investigate the Effects and Mechanism of Astragalus Flavonoids Against Hepatic Fibrosis
Source: Front Pharmacol. 2021 Jan 22;11:618262. doi: 10.3389/fphar.2020.618262 (PMC7862122; doi:10.3389/fphar.2020.618262)
Supplement: Supplementary file 1 [file datasheet1.docx]

**Supplementary Table 1.** Basic information on the five AR flavonoids.

| Molecular ID | Molecule name | Oral bioavailability (OB, %) | Drug likeness (DL) | Molecular weight (g/mol) |
| --- | --- | --- | --- | --- |
| MOL000392 | Formononetin | 69.67 | 0.21 | 268.28 |
| MOL000354 | Isorhamnetin | 49.6 | 0.31 | 316.28 |
| MOL000417 | Calycosin | 47.75 | 0.24 | 284.28 |
| MOL000098 | Quercetin | 46.43 | 0.28 | 302.25 |
| MOL000422 | Kaempferol | 41.88 | 0.24 | 286.25 |

**Supplementary Figure 1.** Schematic drawing to illustrate the IL-17 signaling pathway involving the flavonoids against liver fibrosis. The key targets involved in this process are colored in red.

**
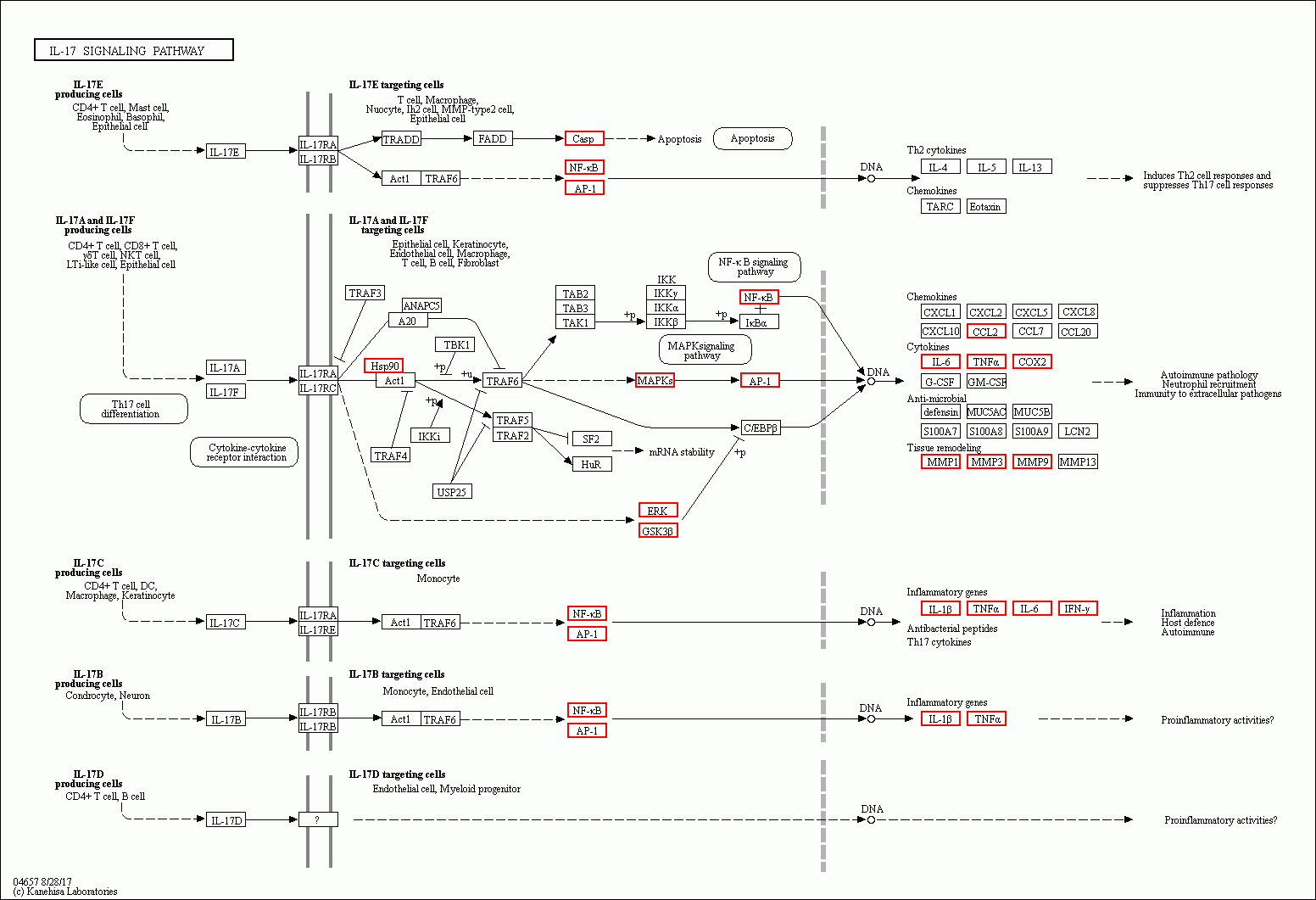
**
